# Supplementary material for: Cdc42 Couples T Cell Receptor Endocytosis to GRAF1-Mediated Tubular Invaginations of the Plasma Membrane
Source: Cells. 2019 Nov 4;8(11):1388. doi: 10.3390/cells8111388 (PMC6912536; doi:10.3390/cells8111388)
Supplement: Supplementary file 1 [file cells-08-01388-s001.zip › cells-631210-supplementary/cells-631210-Supplements.pdf]

Cdc42 couples T cell receptor endocytosis to GRAF1-mediated tubular invaginations of the plasma membrane

Pascal Rossatti et *al.*

SUPPLEMENTAL INFORMATION

Supplemental Figure S1

A

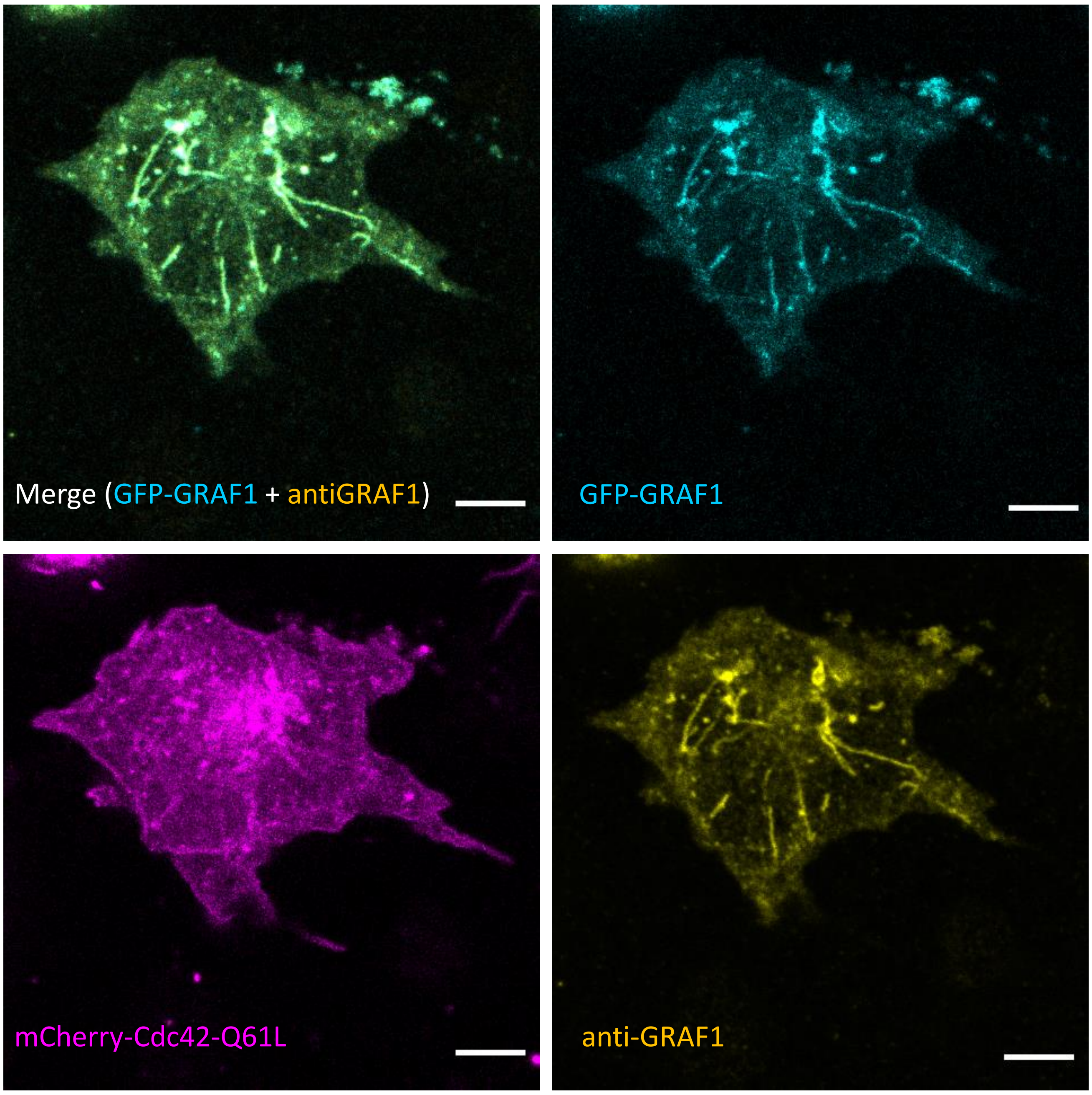

B

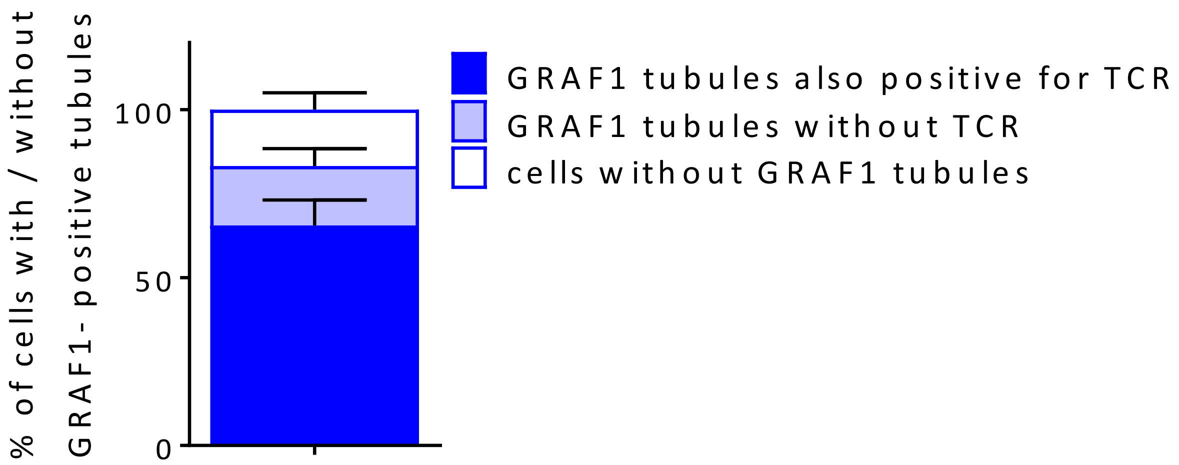

Supplemental Figure S2

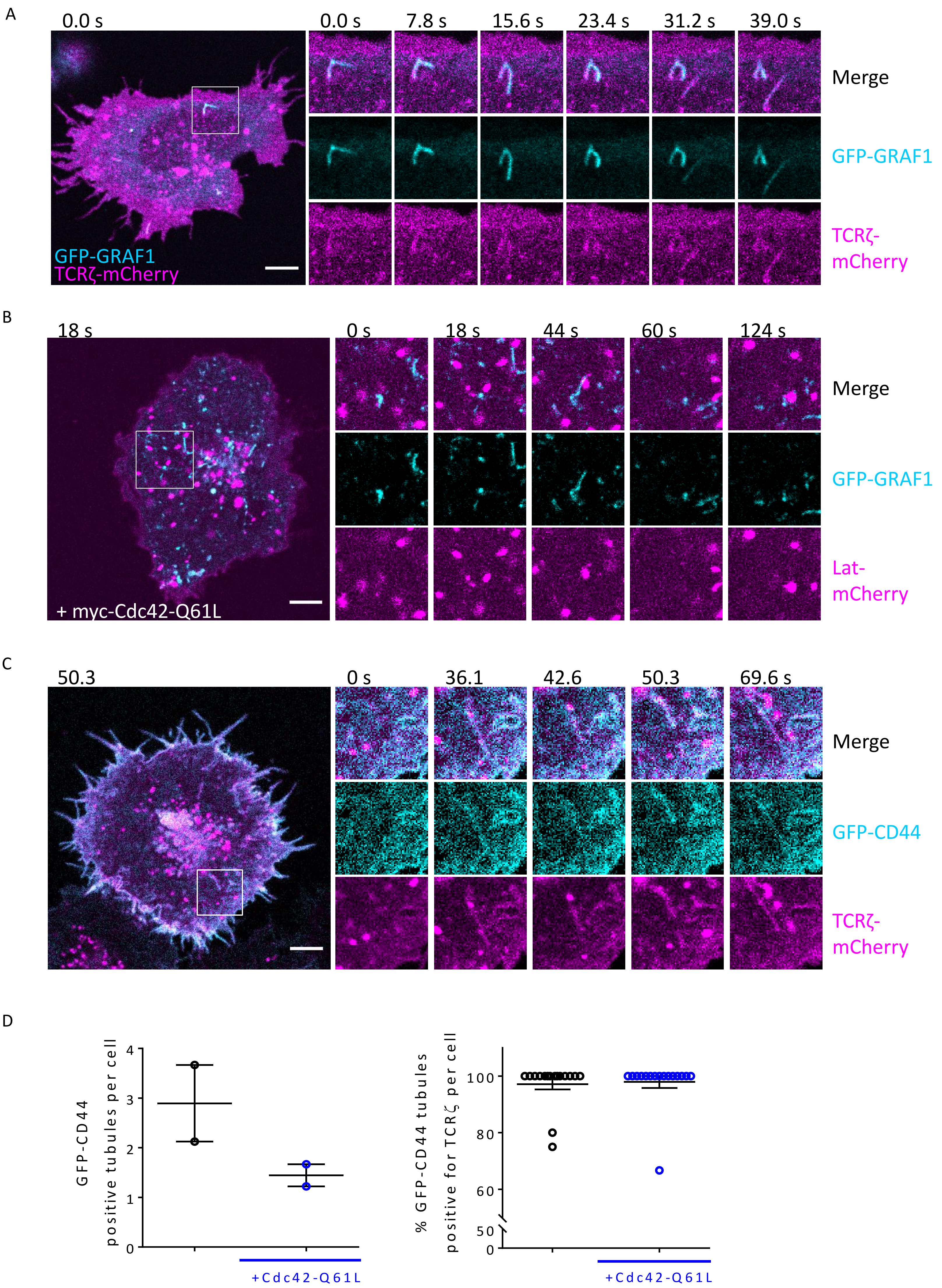

Supplemental Figure S3

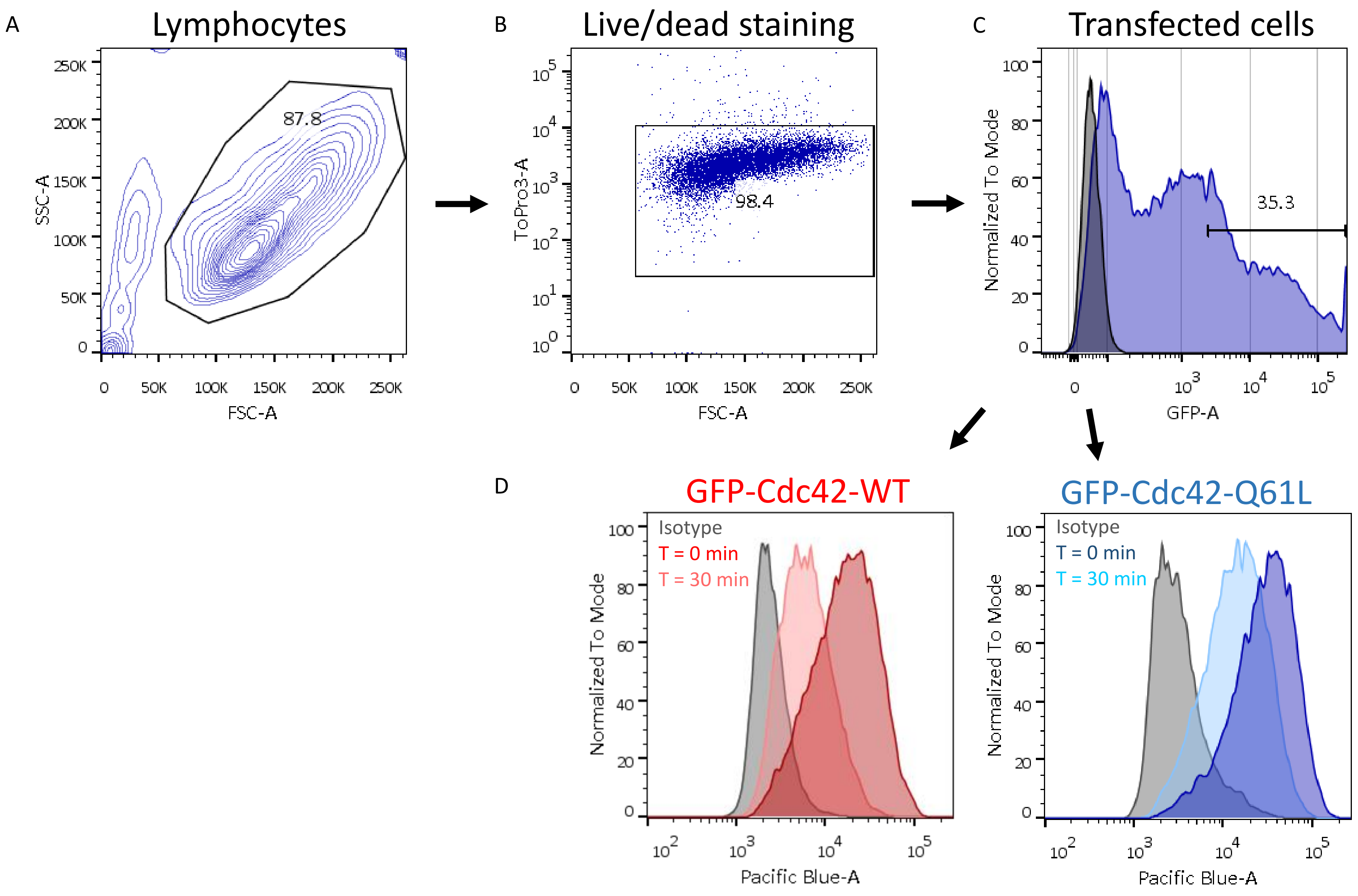

Supplemental Figure S4

A

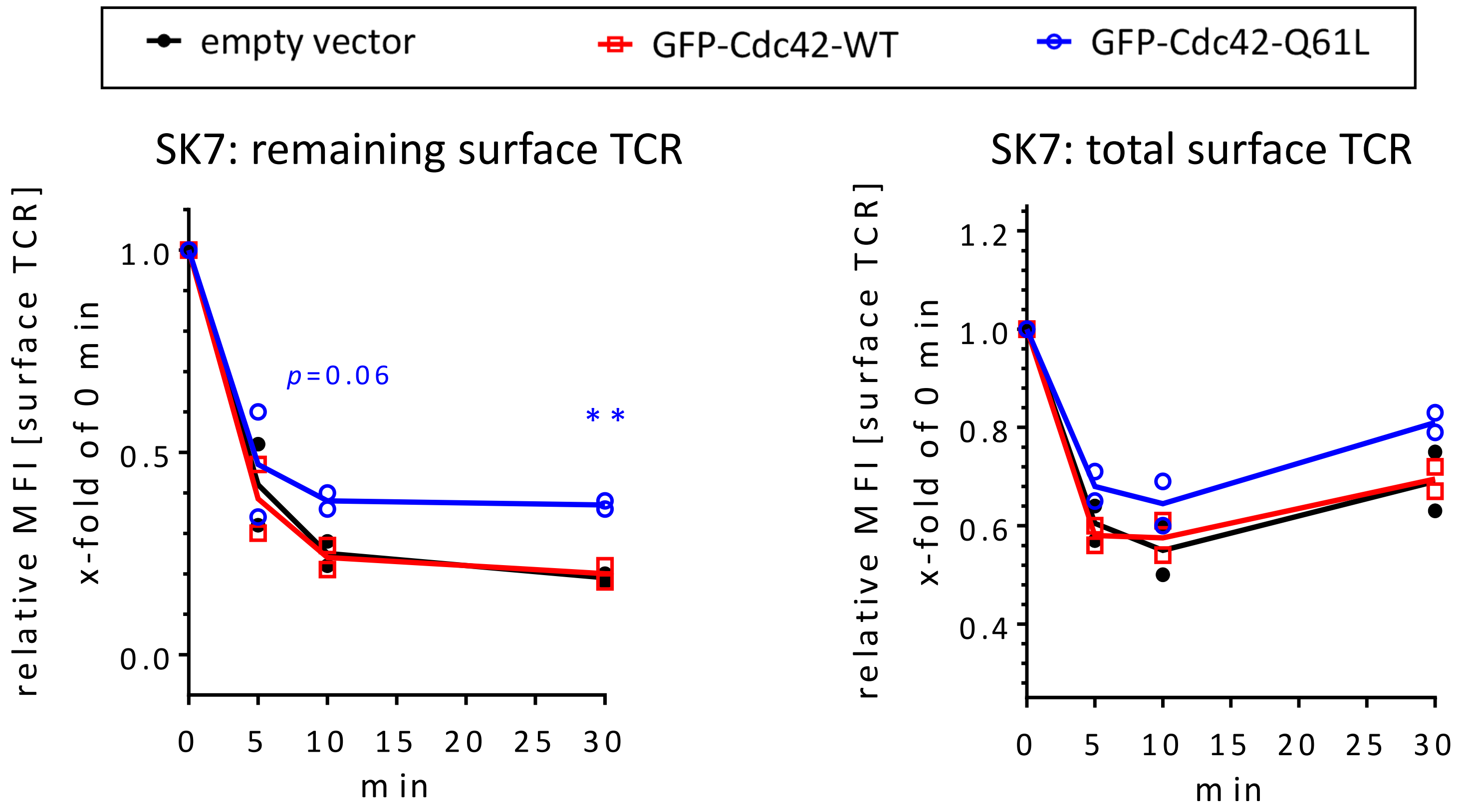

B

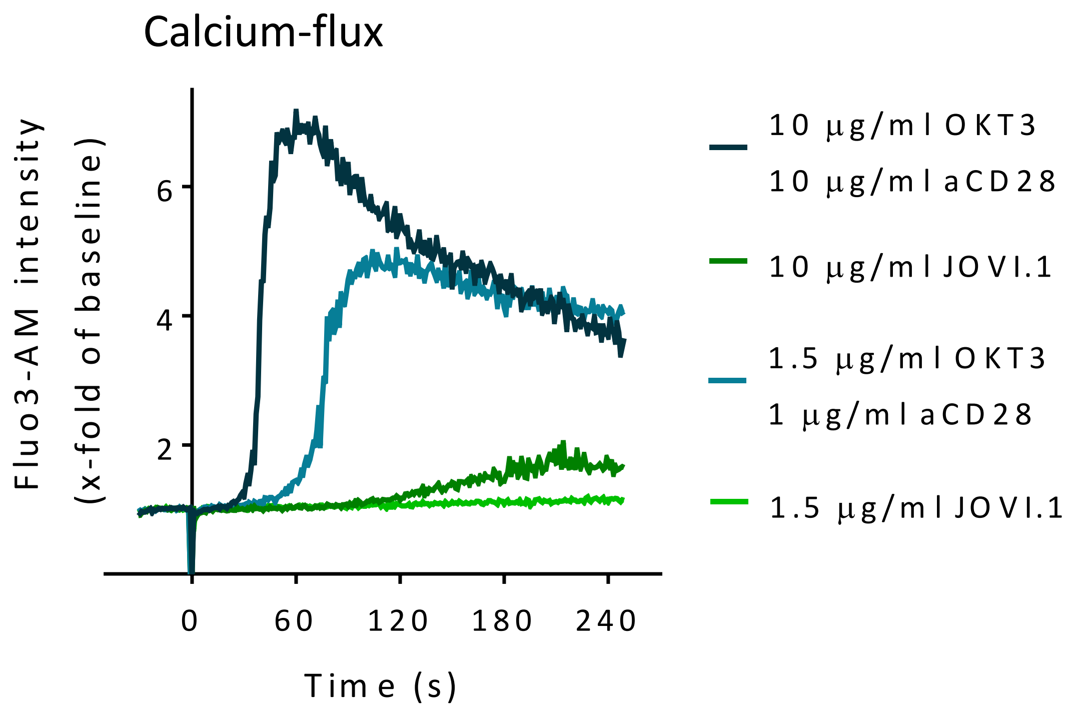

Supplementary Materials

Supplemental Figure S1. (**A**) Commercial anti-GRAF1 labels GFP-GRAF1-positive tubules. Jurkat T cells expressing GFP-GRAF1 and mCherry-Cdc42-Q61L were activated on coverslips coated with functional antibodies against CD3ε and CD28 for 10 min and fixed with 3.7% PFA. GFP-GRAF1 tubules were fully detected by immunostaining using a commercial antibody against GRAF1. Images representative maximum intensity projections of at least three independent experiments. Scale bar: 5 µm. (**B**) Quantification of the number of Jurkat T cells transfected with TCRζ-mCherry, GFP-GRAF1 and myc-Cdc42-Q61L that displayed at least one GRAF1 positive tubule containing TCRζ, or only GRAF1 positive tubules with absent TCRζ-mCherry signal, or no GRAF1 positive tubules at all.

Supplemental Figure S2. (**A**) Representative time series of Jurkat T cells expressing GFP-GRAF1 and TCRζ-mCherry that were activated on coverslips coated with functional antibodies against CD3ε and CD28 and imaged live at 37˚C. (**B**) Representative time series of Jurkat T cells expressing myc-Cdc42-Q61L, GFP-GRAF1 and Lat-mCherry that were activated and imaged as in A. (**C**) Representative time series of Jurkat T cells expressing GFP-CD44 and TCRζ-mCherry that were activated and imaged as in A (**D**) Quantification of the cells as shown in C and with or without expression of Cdc42-Q61L (n=23 and 30 cells from 2 independent experiments). Small horizontal lines indicate mean (±SEM). Scale bar: 5 µm.

Supplemental Figure S3. Gating strategy for the flow cytometry based TCR/Tf internalisation assay. (**A**) Lymphocytes were gated based on FCS/SSC. (**B**) TO-PRO-3 positive (dead) cells were excluded and (**C**) GFP-positive (transfected) cells were selected. (**D**) For transfected cells Pacific Blue was plotted as histogram and mean fluorescence intensity was determined. Note higher Pacific Blue-MFI of cells expressing GFP-Cdc42-Q61L (dark blue) compared to GFP-Cdc42-WT (dark red) and impaired shift of the peak at 30 min in cells expressing GFP-Cdc42-Q61L (light blue) compared to GFP-Cdc42-WT (light red).

Supplemental Figure S4. (A) Remaining TCR-CD3 at the cell surface detected by an antibody against CD3*ε* (clone SK7) after activation-induced internalisation in cells expressing an empty vector, GFP-WT-Cdc42 or GFP-Cdc42-Q61L. (**B**) Flow cytometry measurements of calcium flux based on Fluo-3-AM fluorescence in Jurkat T cells stimulated with soluble antibodies (anti-CD3ε, clone OKT3 and anti-CD28 or anti TCRβ, clone JOVI.1) at the indicated concentrations. Data shown are representative of two independent experiments.

Video S1. Related to Fig2A: Jurkat T cell expressing myc-Cdc42-Q61L, GFP-GRAF1 and TCRζ-mCherry that was activated on coverslips coated with functional antibodies against CD3ε and CD28 and imaged live at 37˚C. Scale bar: 5 µm.

Video S2. Related to Fig2B: Jurkat T cell expressing myc-Cdc42-Q61L and GFP-GRAF1, incubated with Alexa546-Tf for 10 min and imaged live on a coverslip coated with functional antibodies against CD3ε and CD28 at 37˚C, as shown in Fig2B. Scale bar: 5 µm.

Video S3. Related to Fig2D: Dynamics of the TCRζ-PAmCherry signal before, during and after photoactivation in a Jurkat T cell expressing myc-Cdc42-Q61L, GFP-GRAF1 and TCRζ-PAmCherry activated on an antibody coated coverslip and imaged at 37°C with TIRF microscopy, as shown in Fig2D. Scale bar: 5 µm.
